# Multilevel Longitudinal Analysis of Sex Differences in Height Gain and Growth Rate Changes in Japanese School-Aged Children

**DOI:** 10.2188/jea.JE20120164

**Published:** 2013-07-05

**Authors:** Wei Zheng, Kohta Suzuki, Hiroshi Yokomichi, Miri Sato, Zentaro Yamagata

**Affiliations:** 1 Department of Health Sciences, Interdisciplinary Graduate School of Medicine and Engineering, University of Yamanashi, Chuo, Yamanashi, Japan; 2 Center for Birth Cohort Studies, Interdisciplinary Graduate School of Medicine and Engineering, University of Yamanashi, Chuo, Yamanashi, Japan

**Keywords:** growth pattern, sex, puberty, height gain, growth rate

## Abstract

**Background:**

Determining standard pubertal growth patterns using longitudinal anthropometric measures is important in growth assessment. We used an appropriate repeated-measurements method to identify height growth patterns in Japanese school-aged girls and boys.

**Methods:**

The participants were children born during the period from 1991 through 1999 who had entered the first grade of elementary school in the Enzan district in Koshu City, Japan. This study was part of the Project Koshu cohort study. Height was measured annually in April from the first grade of elementary school (age, 6–7 years) to the third grade of junior middle school (age, 14–15 years). Height gain and growth rate trajectories in boys and girls were constructed using multilevel analysis.

**Results:**

In total, 1984 children (1036 boys and 948 girls) were included in this study. Height in boys and girls was similar at age 6.5 to 9.5 years. Girls subsequently grew faster and were taller than boys at age 10.5 to 11.5 years. Starting at age 12.5 years, male height caught up and exceeded female height. Height gain trajectories showed that annual height gain among girls increased slowly and peaked during age 9.5 to 11.5 years, while male height gains declined slightly at first and peaked at age 11.5 to 12.5 years. Sex differences in height gains were significant during the period from age 7.5 to 14.5 years (*P* < 0.0001). Growth rate and height gain trajectories were similar between sexes.

**Conclusions:**

Sex differences in growth trajectory were significant, and female height gain peaked approximately 2 years earlier than male height gain.

## INTRODUCTION

It is important to use anthropometric measures to determine standard growth curves when assessing childhood growth. Height in particular is an important indicator of puberty because of the association between these 2 factors.^1^ In addition, it is generally agreed that height growth differs between sexes. Previous studies showed that male and female height gains differed greatly during the pubertal growth spurt.^2^ Age at height take-off and at peak height velocity is later^3^^,^^4^ in boys than in girls. In addition, duration of the pubertal spurt is longer, and growth velocity is higher, for boys than for girls.^5^

The participants of most previous studies of sex differences were born in Europe around the 1950s, and sample sizes in the studies were small. Early studies of secular pubertal growth changes revealed a trend toward earlier pubertal development in both sexes,^6^^,^^7^ although such changes sometimes differed between sexes. Kagawa et al found that Japanese boys had greater height increments (1.6–3.8 cm) than did girls (1.1–3.1 cm) in each decade from 1950 to 2000.^2^ Hence, further studies are needed to identify modern growth patterns.

Although the Japanese government recently conducted a national survey of childhood growth and clarified related sex differences,^8^ that survey was not a cohort study, and it used cross-sectional data for each age group. Therefore, further studies are needed to clarify the differences in growth rates between sexes. Such studies should use prospective individual data from a cohort study because cohort studies better reveal growth trends among children. In addition, it is desirable to use multilevel analyses, such as the analysis of individual growth used in our previous study (which clarified the association between maternal smoking during pregnancy and childhood growth), because data are collected repeatedly.^9^

We used multilevel analysis to characterize sex differences in longitudinal height gain in a relatively large sample of modern Japanese children.

## METHODS

### Participants

The study data were obtained from Project Koshu (formerly Project Enzan), a dynamic, ongoing Japanese community-based prospective cohort study initiated in 1988, when children were at the fetal stage. Details of this project have been described previously.^9^^–^^13^

Children were eligible for study inclusion if they were born between April 1, 1991 and March 31, 1999 in the Enzan area of Koshu City in Yamanashi Prefecture, Japan and had participated in the first-grade elementary school medical check-up. Children for whom data were not collected during the first grade of elementary school were excluded from the analyses.

This study was approved by the ethical review board of the University of Yamanashi, School of Medicine and was conducted in accordance with the Guidelines Concerning Epidemiological Research (Ministry of Education, Culture, Sports, Science and Technology and Ministry of Health, Labour and Welfare, Japan). The Koshu City administrative office collaborated with the authors of this study.

### Measurements

For the analysis, height was measured annually in April from the first grade of elementary school (average age, 6.5 years) to the third grade of junior high school (average age, 14.5 years), using a stadiometer accurate to 0.1 cm.

### Statistical analyses

Height gain is generally used to compare growth between sexes.^2^^,^^14^ However, because certain height gains may have different meanings for children of different heights, we also calculated growth rate (growth rate = annual height gain/height at the beginning of the year) to adjust for the effect of body size.

The individual growth analysis method (SAS Proc Mixed; SAS Institute Inc, Cary, NC, USA) was used to compare sex differences in annual height gain and growth rate in our study. Because the parametric models used in earlier studies have drawbacks in fitting the growth curve,^15^ we constructed the following model to describe pubertal growth by age period alone, without assuming that the growth curve corresponded to any mathematical function: *E* (height gain*_it_* or growth rate*_it_*) = β_1_ + β_2_ × age period*_it_* + β_3_ × sex*_it_* + β_4_ × age period*_it_* × sex*_it_* + *e_it_* (*i* represents individual, *t* represents time, β_1–4_ represent estimates, and *e* is an error term).

Two main effects (sex and age period) are of interest. If the sex effect is not homogeneous across ages, the interaction term “sex × age period” is added to the model. Details of this analysis were described previously.^9^ All analyses were conducted using SAS version 9.2 (SAS Institute Inc).

## RESULTS

In total, 1984 children (1036 boys and 948 girls) were included in this study. Significant differences in follow-up rates were observed in the age group 12.5 years (Table 1).

**Table 1.** Comparison of height and follow-up rate by sex

Average age (y)	Boys				Girls				*P* value for comparison of follow-up rate^a^
	
	*n*	Follow-up rate (%)	Means (cm)	SD	*n*	Follow-up rate (%)	Means (cm)	SD	
6.5	1036		116.11	5.03	948		115.72	4.91	
7.5	1034	99.8	122.00	5.30	948	100.0	121.60	5.14	0.50
8.5	1036	100.0	127.63	5.67	946	99.8	127.46	5.59	0.23
9.5	1035	99.9	132.91	5.87	947	99.9	133.54	6.19	1.00
10.5	1030	99.4	138.19	6.35	942	99.4	140.27	6.82	0.88
11.5	1019	98.4	144.28	7.39	924	97.5	146.98	6.72	0.16
12.5	995	96.0	151.66	8.38	885	93.4	152.26	5.89	0.01
13.5	986	95.2	158.68	8.26	891	94.0	155.13	5.39	0.24
14.5	732	70.7	164.38	6.89	677	71.4	156.68	5.28	0.71

^a^*P* values were calculated using the χ^2^ test.

### Height by sex

Annual heights from age 6.5 to 14.5 years in both sexes are shown in Table 1. Height was similar between sexes until age 10.5 years, when growth spurts began in girls. Male height caught up at age 12.5 years and exceeded that of girls thereafter.

### Sex differences in height gains and growth rate trajectories

**Figure 1.**
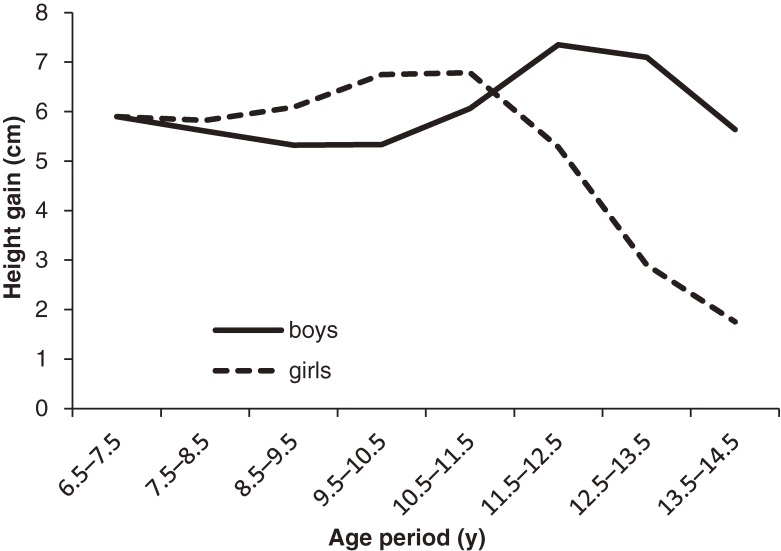
Height gain trajectories in girls and boys, calculated using analyses of individual growth

**Figure 2.**
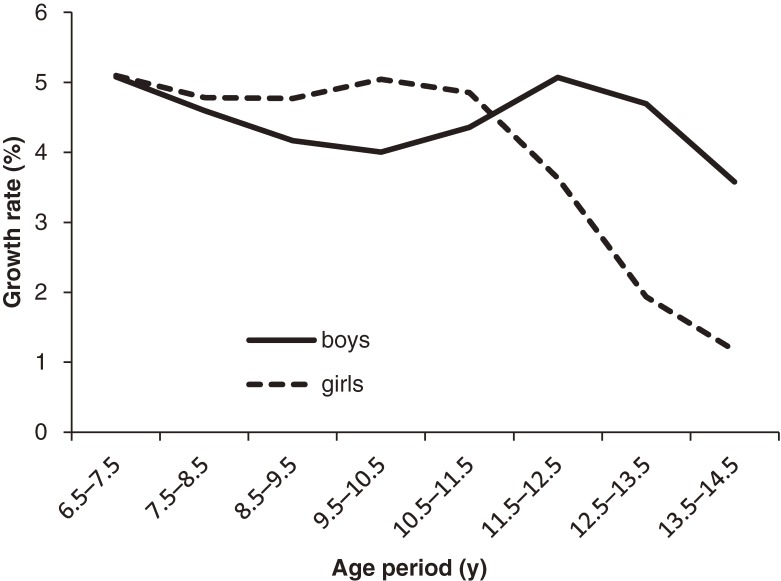
Growth rate trajectories in girls and boys, calculated using analyses of individual growth

Results of analyses of individual growth in boys and girls are shown in Table 2, Figure 1, and Figure 2. As indicated in Table 2, height gain between age 6.5 and 7.5 years was similar between sexes. However, in all subsequent age periods, sex differences in height gain were significant, as demonstrated by the interaction term “sex × age period.” As shown in Figure 1, male annual height gains decreased slightly from age 6.5 to 10.5 years. The growth spurt then started in boys and peaked between age 11.5 and 12.5 years. However, female height gains showed a steady but slowly increasing trend from age 7.5 years and peaked between age 9.5 and 11.5 years. As compared with height gain trajectories, growth rate trajectories showed similar sex differences across ages (Figure 2). No sex difference in growth rate was found between age 6.5 years and 7.5 years. However, the interaction terms indicated that the sex effect was not homogeneous across subsequent age periods (Table 2).

**Table 2.** Solution for fixed effect of height gain and growth rate by age period, sex, and their interaction

	Height gain			Growth rate		
	
	Estimate	SE	*P* value	Estimate	SE	*P* value
Intercept	5.88	0.03	<0.0001	5.07	0.03	<0.0001
7.5–8.5 y	−0.24	0.05	<0.0001	−0.44	0.04	<0.0001
8.5–9.5 y	−0.62	0.07	<0.0001	−0.94	0.05	<0.0001
9.5–10.5 y	−0.59	0.07	<0.0001	−1.09	0.05	<0.0001
10.5–11.5 y	0.16	0.07	0.02	−0.71	0.05	<0.0001
11.5–12.5 y	1.51	0.09	<0.0001	0.04	0.06	0.48
12.5–13.5 y	1.13	0.10	<0.0001	−0.41	0.07	<0.0001
13.5–14.5 y	−0.12	0.10	0.24	−1.36	0.07	<0.0001
Girls	−0.002	0.04	0.96	0.02	0.04	0.57
7.5–8.5 y × girls	0.22	0.08	0.004	0.18	0.06	0.007
8.5–9.5 y × girls	0.81	0.10	<0.0001	0.62	0.08	<0.0001
9.5–10.5 y × girls	1.39	0.09	<0.0001	1.01	0.07	<0.0001
10.5–11.5 y × girls	0.66	0.11	<0.0001	0.43	0.08	<0.0001
11.5–12.5 y × girls	−2.20	0.12	<0.0001	−1.56	0.09	<0.0001
12.5–13.5 y × girls	−4.13	0.15	<0.0001	−2.77	0.10	<0.0001
13.5–14.5 y × girls	−4.23	0.15	<0.0001	−2.74	0.11	<0.0001

Abbreviation: SE, standard error.

## DISCUSSION

This study focused on sex differences in height growth among Japanese children and is the first to use multilevel analysis to examine growth trajectories at the population level in girls and boys. We found that height gains among girls increased steadily from age 7.5 years and peaked between age 9.5 and 11.5 years, whereas height gains among boys showed a decreasing trend until age 10.5 years. Growth spurts then started in boys and peaked between age 11.5 and 12.5 years. Differences in growth patterns were also reflected in absolute standing height.

We used multilevel analysis to investigate growth trajectories at the population level. Many previous studies fit the growth curve at the individual level because they included only a small number (dozens) of participants. The large sample size of the present study makes its results more reliable. However, growth trajectory at the population level requires analytic methods that consider correlation and variation both within and among individuals. The standard method—repeated-measures analysis of variance—cannot fit the time-dependent independent covariates and cannot flexibly handle missing data. In contrast, multilevel analysis can fit a population-level model that considers each individual’s growth trajectory. Therefore, its use is appropriate for this study.

The average heights of boys and girls from the first grade of elementary school to the third grade of junior high school were similar those described in a Japanese national survey in 2011, which implies that that our sample of Japanese children is representative.^8^

The trajectories showed that male height gains declined until age 10.5 years, while female height gains declined only slightly at age 7.5 to 8.5 years, before the pubertal growth spurt occurred. The previously described mid-growth spurt around age 7 years may explain these decreases,^16^ but the absence of height data before age 6.5 years prevented us from analyzing the entire mid-growth spurt.

Pubertal growth spurts were seen earlier in girls than in boys, which gave girls a significant height advantage at age 10.5 to 11.5 years. Starting at age 11.5 years, boys had a greater increase in height. This difference is similar to the results of a recent cross-sectional study by Zivicnjak et al in Croatia^2^ and those of the Japanese national survey.^8^ However, another study, conducted by Hauspie et al in India in the 1950s, showed that girls were slightly taller than boys at age 11.0 to 14.0 years, suggesting a longer delay in the growth spurt among boys as compared with girls.^4^

Our results regarding sex differences in stature were consistent with those for height gain. Our findings suggest that height gains among girls were greater until age 11.5 years (height gain peaked at age 9.5–11.5 years), at which point an intersection of the 2 height gain curves, caused by the greater increment in male height gain, occurred at approximately age 11.5 years (height gain peaked at age 11.5–12.5 years). This finding is in good agreement with those of Zivicnjak et al.^2^ However, the results of the study of Indian children showed that, starting at age 13.0 years, male height gains exceeded female height gains.^4^ Another study that combined 6 cross-sectional studies in Greece found that girls reached their height-gain peak mostly between age 11 to 12 years and that male annual height gain exceeded female annual height gain thereafter and peaked mainly during age 14 to 15 years.^17^ The delay in height gain peak in Indian children and Greek children may be due to differences in racial characteristics. However, the early periods of surveillance (1952–1966 in India and 1928–1995 in Greece) may also account for the difference. As compared with the results of our study, the peak annual difference in median height (similar to annual height gain in our study), as described in the World Health Organization Multicentre Growth Reference Study, was 1 year later in boys but at almost the same age in girls.^18^

Few studies have analyzed the interaction between sex and age. Our study mainly focused on sex differences across ages; therefore, it used height gain curves with annual increments in individual growth analysis and added the interaction term “sex × age period.” Our findings suggest that sex differences were significant at all age periods, except age 6.5 to 7.5 years. A study by Sheehy et al also found that the overall sex effect was not homogeneous across developmental phases.^5^ However, their study did not refer to the sex effect of each age phase, due to methodological limitations in their analysis.

Growth rate trajectories focus on sex differences in growth patterns by controlling for sex and individual differences in body size. In this study, the results of growth rate trajectories were consistent with those of height gain trajectories, as described above, suggesting that the effect of body size did not influence sex differences in growth pattern.

This study has some limitations. First, because height was measured in April, age was inconsistent among participants. However, height was measured at the same time in both sexes. As such, the effect of age variation on ascertainment of growth differences between sexes is likely to be small. Second, follow-up did not continue until participants were fully grown. Nevertheless, the purpose of this study was to explore sex differences in growth. A marked decline was seen in the latter half of the height gain and growth rate trajectories in both sexes. Thus, differences between sexes from pre-puberty to peak velocity could be clarified using the current data. Finally, follow-up rates declined markedly in the last year of the study because annual height measurement collection was suspended in 2007. However, follow-up rates were consistent between sexes.

In conclusion, analysis of individual growth showed that sex differences in growth patterns were significant. However, our results only describe population-level growth among modern Japanese children. Further studies on growth patterns are needed, since pubertal growth varies according to time and ethnicity.
